# Clinical characteristics of 145 patients with corona virus disease 2019 (COVID-19) in Taizhou, Zhejiang, China

**DOI:** 10.1007/s15010-020-01432-5

**Published:** 2020-04-28

**Authors:** Qingqing Chen, Zhencang Zheng, Chao Zhang, Xijiang Zhang, Huijuan Wu, Jingdong Wang, Shuwei Wang, Cheng Zheng

**Affiliations:** 1 Department of Critical Care Medicine, Taizhou Enze Medical Center (Group) Enze Hospital, Taizhou, 318050 Zhejiang China; 2 Department of Critical Care Medicine, Taizhou Municipal Hospital, Taizhou, 318000 Zhejiang China; 3 Department of Critical Care Medicine, Taizhou Enze Medical Center (Group) Luqiao Hospital, Taizhou, 318050 Zhejiang China

**Keywords:** Corona virus disease 2019, COVID-19, SARS-CoV-2, Clinical characteristics, Epidemiology, Treatment, Outcomes

## Abstract

**Objective:**

The aim of this study was to investigate the clinical characteristics of Corona Virus Disease 2019 in Taizhou, China.

**Methods:**

A single center retrospective observational study was performed between Jan 1, 2020 and Mar 11, 2020 at Taizhou Public Health Medical Center, Zhejiang, China. All patients with confirmed Corona Virus Disease 2019 were enrolled, and their clinical data were gathered by reviewing electronic medical records. Outcomes of severely ill patients and non-severely ill patients were compared.

**Results:**

Of 145 hospitalized patients with COVID-19, the average age was 47.5 years old (standard deviation, 14.6) and 54.5% were men. Hypertension was the most common comorbidity (15.2%), followed by diabetes mellitus (9.7%). Common symptoms included dry cough (81.4%), fever (75.2%), anorexia (42.8%), fatigue (40.7%), chest tightness (32.4%), diarrhea (26.9%) and dizziness (20%). According to imaging examination, 79.3% patients showed bilateral pneumonia, 18.6% showed unilateral pneumonia, 61.4% showed ground-glass opacity, and 2.1% showed no abnormal result. Compared with non-severely ill patients, severely ill patients were older (mean, years, 52.8 vs. 45.3, *p* < 0.01), had a higher proportion of diabetes mellitus (16.3% vs. 6.9%, *p* = 0.08), had a higher body mass index (mean, 24.78 vs. 23.20, *p* = 0.02) and were more likely to have fever (90.7% vs. 68.6%, *p* = 0.01), anorexia (60.5% vs. 35.3%, *p* = 0.01), chest tightness (60.5% vs.20.6%, *p* < 0.01) and dyspnea (7.0% vs. 0%, *p* = 0.03). Of the 43 severely ill patients, 6 (14%) received high-flow nasal cannula oxygen therapy, and 1 (2.3%) received invasive mechanical ventilation.

**Conclusions:**

Older patients or patients with comorbidities such as obesity or diabetes mellitus were more likely to have severe condition. Treatments of COVID-19 is still experimental and more clinical trials are needed.

## Introduction

In early December 2019, a group of acute respiratory illness, now known as Corona Virus Disease 2019 (COVID-19) occurred in Wuhan, Hubei Province, China [1–5]. The disease has spread rapidly from Wuhan to other parts of China and even around the world. The new novel coronavirus was identified in samples of airway epithelial cells from a patient in Wuhan and was confirmed as the cause of COVID-19 [6]. After a month, the Coronavirus Study Group of the International Committee on Taxonomy of Viruses designates it as severe acute respiratory syndrome coronavirus 2 (SARS-CoV-2) [7]. As with the closely related severe acute respiratory syndrome (SARS) coronavirus, there is evidence of human-to-human transmission, extensively in family settings, but also in hospitals [8–13].

As of April 13th, 2020, a total of 82,249 COVID-19 cases in China have been confirmed with 3341 deaths [14]. Internationally, cases have been reported in 213 countries, areas or territories [15]. At present, information regarding the epidemiology and clinical features of COVID-19 is scarce. Taizhou is a prefecture level city of Zhejiang Province with a population of 6 million. However, Taizhou has a large number of people engaging in trade and learning in Wuhan. According to statistics alone, there were more than 20,000 people returning from Wuhan to Taizhou when the epidemic started. Therefore, Taizhou has become one of the main outbreak places of imported cases. Given the rapid spread of SARS-CoV-2, an analysis with larger sample size cases in Taizhou is urgently warranted. Here, by collecting the data from 145 laboratory-confirmed cases, we sought to provide an up-to-date delineation of the clinical characteristics of patients with COVID-19 throughout Taizhou. The objective of this case series was to describe the clinical characteristics of 145 hospitalized patients with COVID-19 and to compare severely ill patients with non-severely ill patients.

## Materials and methods

### Patients and study design

The study was approved by the Ethics Committee of Taizhou Enze Medical Center (Group) Enze Hospital (No. K20200303). Due to the retrospective nature of the study, the Ethics Committee determined that no patient consent was required. In addition, a statement of permission from patients for submission the present study was not required as the study did not include any personal information.

Taizhou Public Health Medical Center is located in Taizhou Enze Medical Center (Group) Enze Hospital, Zhejiang Province, which is jointly established by Taizhou Municipal Government and Taizhou Enze Medical Center. As the first special medical institution for infectious diseases in Taizhou, it is responsible for the task issued by Zhejiang Provincial Government in the treatment of COVID-19 in Taizhou. According to the arrangements put in place by the Zhejiang Provincial Government, all patients were admitted centrally to the hospital from the whole Taizhou without selectivity. All patients with COVID-19 enrolled in this study were diagnosed according to World Health Organization interim guidance [16]. The clinical outcomes (ie, discharges, length of stay) were monitored up to March 11th, 2020.

### Data collection

The medical records of patients were analyzed by the team of the Department of Critical Care Medicine, Taizhou Enze Medical Center (Group) Enze Hospital. The patients’ data were collected by reviewing electronic medical records. We recorded demographic data including age and gender, the clinical data including underlying diseases, medical history, exposure history, symptoms, signs, laboratory findings, chest computed tomographic (CT) scans, and treatment measures (ie, antiviral therapy, corticosteroid therapy, respiratory support), Sequential Organ Failure Assessment (SOFA) score, MuLBSTA score, the Acute Physiology and Chronic Health Evaluation (APACHE) II, epidemiological, and outcomes data.

### Statistical analysis

Statistical analysis was performed with SPSS 20.0 software (IBM Corp, Armonk, NY, USA). Continuous variables were presented as mean ± standard deviation if normally distributed, and as median and interquartile range (IQR) if non-normally distributed. Continuous variables were compared by Student t test or Mann–Whitney *U* test and enumeration variables were compared by Pearson *χ*^2^ or Fisher exact test, where appropriate. A two-tailed *P* < 0.05 was considered statistically significant. The analyses have not been adjusted for multiple comparisons and, given the potential for type I error, the findings should be interpreted as exploratory and descriptive.

### Definitions

Cases were diagnosed based on the World Health Organization (WHO) interim guidance [16]. A confirmed case with COVID-19 was defined as a positive result to high-throughput sequencing or real-time reverse-transcriptase polymerase-chain-reaction (RT-PCR) assay for nasal and pharyngeal swab specimens [17]. Acute respiratory distress syndrome (ARDS) was defined according to the Berlin definition [18]. For severely and non-severely ill patients, refer to Diagnosis and Treatment of Pneumonia caused by SARS-CoV-2 (version 7) [19] issued by of National Health Commission of the People's Republic of China. Severe condition is defined as one of the following: (1) The respiratory rate is more than 30 times/min; (2) In the resting state, transcutaneous oxygen saturation (SaO_2_) ≤ 93%; (3) Oxygenation index (PaO_2_/FiO_2_) ≤ 300 mmHg. Sepsis was defined according to the new definition of Sepsis-3 [20].

## Results

### Demographic and clinical characteristics

The study population included 145 hospitalized patients with confirmed COVID-19. The demographic and clinical characteristics of these patients were summarized in Table 1. The average age was 47.5 years old (S.D. 14.6), and 54.5% (79/145) were male. All patients were admitted to the isolation ward for treatment, including 43 severe cases. One patient was admitted to intensive care. Hypertension was the most common comorbidity (15.2%, 22/145), followed by diabetes mellitus (9.7%, 14/145). The average age of severely ill patients was older than that of non-severely ill patients (mean, years, 52.8 vs. 45.3, *p* < 0.01), and the body mass index (BMI) of severely ill patients was higher than non-severely ill patients (mean, 24.78 vs. 23.20, *p* = 0.02), but there were no significant differences in smoking history and gender between the two groups. In terms of co-morbidities, a significant high percentage of diabetes mellitus was observed in severely ill patients (16.3% vs. 6.9%, *p* = 0.08). As expected, severely ill patients had a higher APACHE II score (median, 5 vs. 3, *p* < 0.01), a higher SOFA score (median, 2 vs. 1, *p* < 0.01) and a higher MuLBSTA score (median, 9 vs. 5, *p* < 0.01). In terms of epidemiology, 45.5% (66/145) of the patients were those who returned to Taizhou from or around Wuhan, 44.8% (65/145) were close contacts, and 9.7% (14/145) could not determine the source.

**Table 1:** Baseline characteristics of patients with COVID-19

Characteristics	Non-severely ill patients (*n* = 102)	Severely ill patients (*n* = 43)	*P*-value
Age, median years (IQR)	45.3 ± 13.6	52.8 ± 15.5	0.00
Male sex	56 (54.9%)	23 (53.5%)	0.88
BMI	23.20 (21.66,25.71)	24.78 (23.07,26.96)	0.02
*Comorbidities*			
Hypertension	13 (12.7%)	9 (20.9%)	0.21
Diabetes mellitus	7 (6.9%)	7 16.3%)	0.08
COPD	6 (5.9%)	0 (0%)	0.18
Chronic liver disease	2 (2.0%)	4 (4.7%)	0.73
Chronic kidney disease	2 (2.0%)	1 (2.3%)	1
Peptic ulcer	1 (1%)	2 (4.7%)	0.21
Solid tumor	1 (1%)	2 (4.7%)	0.44
Chronic cardiac insufficiency	0 (0%)	1 (2.3%)	0.30
HIV infection	0 (0%)	1 (2.3%)	0.30
Hyperlipidemia	0 (0%)	1 (2.3%)	0.30
Smoking history	12 (11.8%)	3 (7.0%)	0.57
*Exposure to source of transmission within 14 days*			
Returned from Wuhan	49 (48%)	17 (39.5%)	0.35
Close contact with the confirmed patient who returned from Wuhan	46 (45.1%)	19 (44.2%)	0.92
Uncertain	7 (6.9%)	7 (16.3%)	0.08
APACHE II score, median (IQR)	3 (1,5)	5 (3,8)	0.00
SOFA score, median (IQR)	1 (0,1.25)	2 (1,3)	0.00
MuLBSTA score, median (IQR)	5 (4.75,7)	9 (7,11)	0.00
*Hospitalization ward*			
ICU stay	0 (0%)	1 (2.3%)	0.30

*IQR* interquartile range, *COPD* chronic obstructive pulmonary disorder, *SOFA* sequential organ failure assessment, *APACHE* acute physiology and chronic health evaluation, *ICU* intensive care unit, *BMI* body mass index

Signs and symptoms of patients with COVID-19 were shown in Table 2. The most common symptoms at onset of illness were dry cough (81.4%, 118/145), fever (75.2%, 109/145), anorexia (42.8%, 62/145), fatigue (40.7%, 59/145), chest tightness (32.4%, 47/145), diarrhea (26.9%, 39/145) and dizziness (20%, 29/145). Less common symptoms were nausea, headache, myalgia, rhino-pharyngitis, abdominal pain, vomiting, dyspnea and hypoacusis (Table 2). Compared with non-severely ill patients, severely ill patients were more likely to report fever, anorexia, chest tightness and dyspnea (90.7% vs. 68.6%, 60.5% vs. 35.3%, 60.5% vs. 20.6% and 7.0% vs. 0%, all *p* < 0.05), and the duration of fever in severely ill patients was longer (median days, 6 vs. 4, *p* < 0.01). The median time from onset of symptoms to hospitalization was 6 days (IQR, 3–9). There was no difference in signs between the two groups.

**Table 2:** Signs and symptoms of patients with COVID-19

Signs and symptoms	Non-Severely ill patients (*n* = 102)	Severely ill patients (*n* = 43)	*P*-value
*Symptoms*			
Dry cough	80 (78.4%)	38 (88.4%)	0.34
Fever	70 (68.6%)	39 (90.7%)	0.01
Anorexia	36 (35.3%)	26 (60.5%)	0.01
Fatigue	38 (37.3%)	21 (48.8%)	0.20
Chest tightness	21 (20.6%)	26 (60.5%)	0.00
Diarrhea	23 (22.5%)	16 (37.2%)	0.07
Dizziness	23 (22.5%)	6 (14%)	0.24
Rhino-pharyngitis	20 (19.6%)	8 (18.6%)	0.89
Nausea	14 (13.7%)	10 (23.3%)	0.16
Headache	16 (15.7%)	8 (18.6%)	0.67
Myalgia	13 (12.7%)	7 (16.3%)	0.57
Abdominal pain	6 (5.9%)	2 (4.7%)	1
Vomiting	3 (2.9%)	3 (7.0%)	0.51
Dyspnea	0 (0%)	3(7.0%)	0.03
Hypoacusis	2 (2.0%)	0 (0%)	1
Duration of fever (days) (IQR)	4 (2,6)	6 (4,8)	0.00
No abnormality was noted on initial presentation	12 (11.8%)	0 (0%)	0.02
Onset of symptom to hospital admission, median (IQR)	5 (3,9)	6 (3,10)	0.23
*Signs*			
Heart rate, median (IQR), bpm	83 (76,90.5)	83 (75,93)	0.81
Respiratory rate, median (IQR)	19 (18,20)	19 (18,20)	0.32
Mean arterial pressure, media (IQR), mmHg	97 (88,104)	98 (90,104)	0.75

*IQR* interquartile range, *bpm* beat per minute

### Laboratory and radiologic parameters in severely and non-severely ill patients

There were numerous differences in laboratory findings between severely ill patients and non-severely ill patients (Table 3). Severely ill patients have higher absolute neutrophil count and erythrocyte sedimentation rate, as well as higher levels of activated partial thromboplastin time, troponin I, creatine kinase, aspartate aminotransferase, gamma glutamyl transpeptidase, lactate dehydrogenase and procalcitonin. In terms of lymphocyte count, albumin, partial pressure of oxygen and carbon dioxide were lower in severely ill patients.

**Table 3:** Radiologic and laboratory findings of patients with COVID-19

Radiologic and laboratory findings	Non-Severely ill patients (*n* = 102)	Severely ill patients (*n* = 43)	*P*-value
*Blood routine test*			
WBC (× 10^9^/L) (IQR)	5 (4.18,6.4)	6 (4.44,7.40)	0.07
Hb(g/L) (mean ± S.D.)	139.78 ± 15.98	133.98 ± 17.35	0.05
ANC (× 10^9^/L) (IQR)	3.1 (2.38,4.4)	4.5 (2.7,5.6)	0.00
Lymphocyte count (× 10^9^/L) (IQR)	1.3 (1,1.63)	0.9 (0.6,1.1)	0.00
Monocyte count (× 10^9^/L) (IQR)	0.4 (0.3,0.5)	0.4 (0.3,0.5)	0.57
Platelet (× 10^9^/L) (IQR)	204.5 (175,254)	192 (142,259)	0.27
CRP (mg/L) (IQR)	2.6 (1,8.6)	4.7 (1,26.78)	0.10
ESR (mm/h) (IQR)	30 (17,45)	42 (30,63)	0.00
*Coagulation function*			
PT (s) (IQR)	11.85 (11.3,12.4)	11.9 (11.45,12.5)	0.27
APTT (s) (IQR)	29.2 (27.63,31.85)	31.2 (28.5,32.8)	0.02
D-dimer (mg/L) (IQR)	0.24 (0.16,0.39)	0.32 (0.21,0.49)	0.11
*Cardiac function*			
CK (μg/L) (IQR)	60 (42.75,79.25)	90 (59,166)	0.00
CK-MB (μg/L) (IQR)	0.72 (0.41,1.25)	0.87 (0.43,2.28)	0.21
TnI (μg/L) (IQR)	0.01 (0,0.01)	0.01 (0.01,0.01)	0.01
*Liver and kidney function*			
Albumin (g/L) (mean ± S.D.)	40.35 ± 3.95	37.20 ± 4.68	0.00
ALT (U/L) (IQR)	20 (14,31.5)	25 (15,37)	0.10
AST (U/L) (IQR)	23.5 (19,30)	28 (20,45)	0.02
ALP (U/L) (IQR)	72 (60.75,87)	68 (56,80)	0.14
γ-GT (U/L) (IQR)	23 (16,37)	32 (21,55)	0.00
LDH (U/L) (IQR)	187 (165.25,183)	241 (207,311)	0.00
TBil (μmol/L) (IQR)	12.75 (8.35,17.38)	14.9 (11,19.2)	0.11
SCr (μmol/L) (IQR)	74 (66,88)	74 (65,93)	0.57
BUN (mmol/L) (IQR)	4 (3.2,4.85)	4.5 (3.1,5.7)	0.15
PCT (ng/mL) (IQR)	0.03 (0.02,0.05)	0.05 (0.03,0.07)	0.01
*Abnormalities on chest CT*			
Ground-glass opacity	60 (58.8%)	29 (67.4%)	0.33
Unilateral pneumonia	25 (24.5%)	2 (4.7%)	0.01
Bilateral pneumonia	75 (73.5%)	40 (93.0%)	0.01
Normal	2 (2.0%)	1 (2.3%)	1
*Blood gas analysis*			
pH (IQR)	7.42 (7.40,7.44)	7.45 (7.42,7.47)	0.00
PaO_2_ (mmHg) (IQR)	93.5 (82.75,111)	72 (65,81)	0.00
PaCO_2_ (mmHg) (IQR)	42 (39,45)	39 (35,41)	0.00
Lactate (mmol/L) (IQR)	1.8 (1.3,2.2)	2.1 (1.4,2.9)	0.55

*IQR* interquartile range, *WBC* white blood count, *ANC* absolute neutrophil count, *ALT* alanine aminotransferase, *AST* aspartate aminotransferase, *ALP* alkaline phosphatase, *γ-GT* gamma glutamyl transpeptidase, *LDH* lactate dehydrogenase, *TBil* total bilirubin, *SCr* serum creatinine, *PCT* procalcitonin, *PT* prothrombin time, *APTT* activated partial thromboplastin time, *CK* creatine kinase, *CK-MB* creatine kinase MB, *CRP* C-reactive protein, *BUN* blood urea nitrogen, *TnI* troponin I, *ESR* erythrocyte sedimentation rate, *CT* computed tomographic, *Hb* hemoglobin, *pH* hydrogen ion concentration

**Fig. 1:**
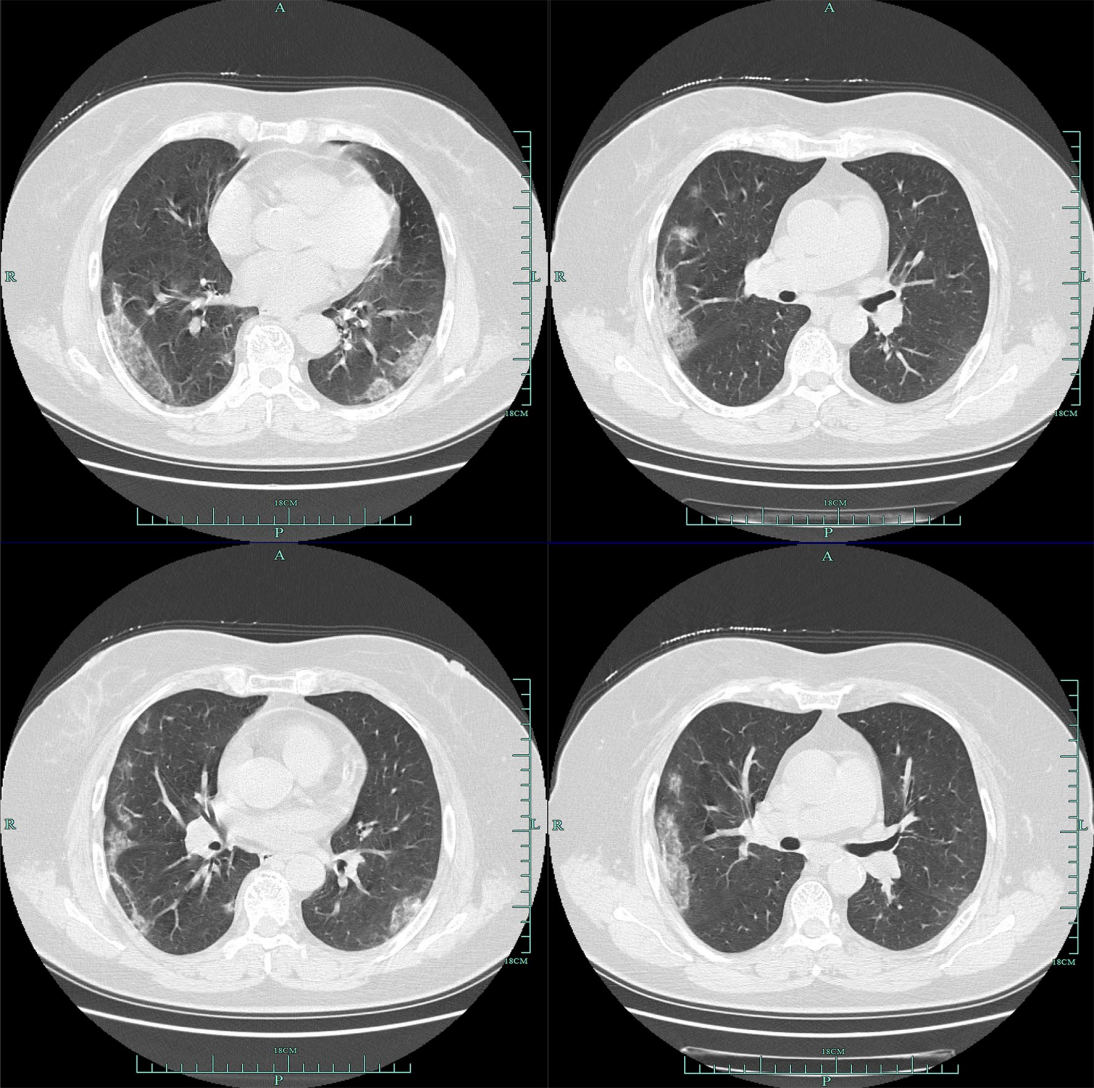
Multiple patchy shadows and ground-glass opacity were observed in both lungs

**Fig. 2:**
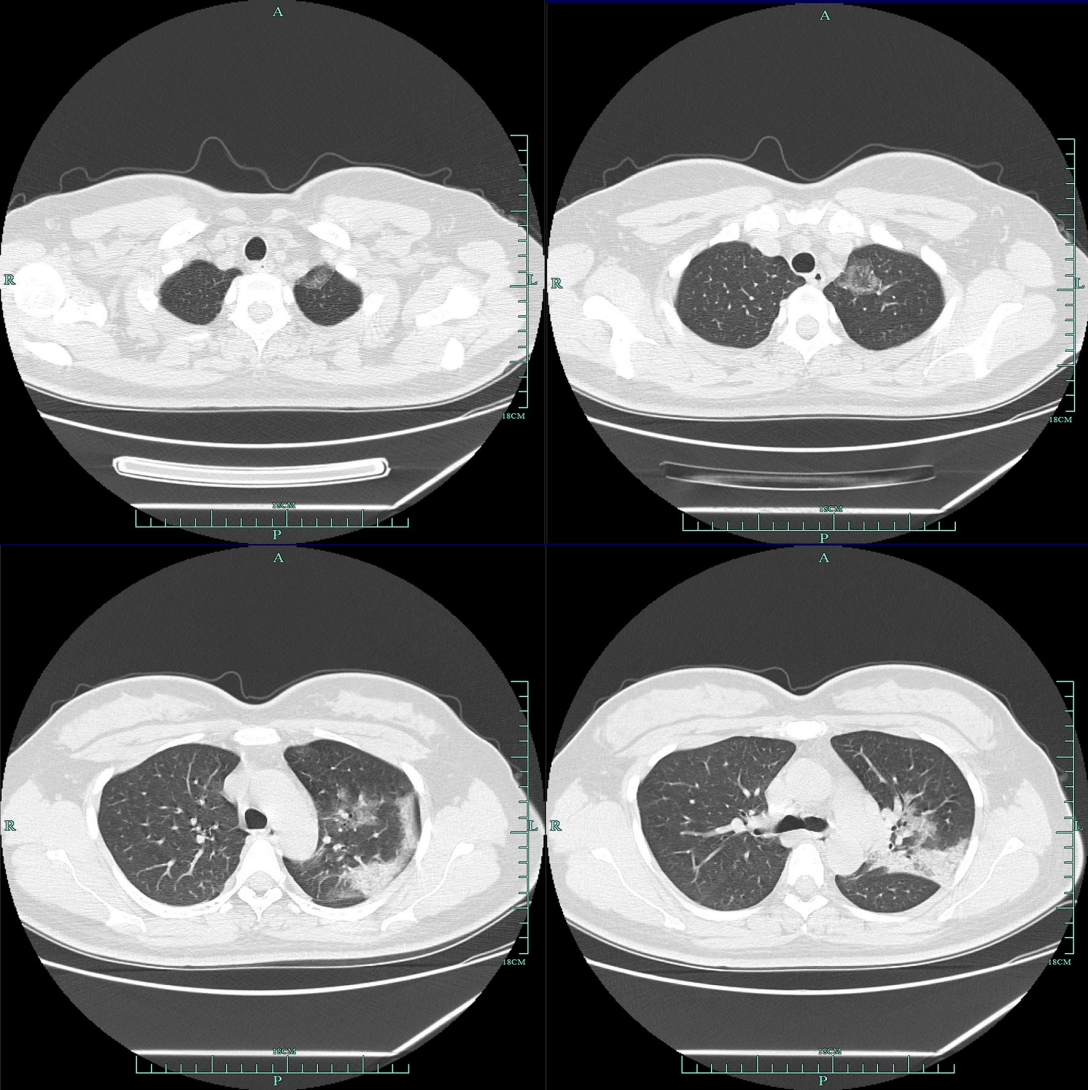
Ground-glass opacity and patchy and patchy high-density shadows were mainly on the left middle lobe and the lower left lobe, edges were blurred, ground-glass opacity were observed in the upper left lobe. A few fibrous high-density shadows were observed in the left lower lobe

According to CT, 79.3% (115/145) patients showed bilateral pneumonia (Fig. 1) with just 18.6% (27/145) patients showing unilateral pneumonia (Fig. 2). 61.4% (89/145) patients showed ground-glass opacity (Table 3). Finally, 2.1% (3/145) patients showed no abnormal results.

### Main intervention measures and outcome

As of March 11, all patients had been discharged. Only one patient has been admitted to ICU and has been discharged. Complications among the 145 patients included sepsis (36.6%, 53/145) and ARDS (0.07%, 1/145). Severely ill patients were more likely to have sepsis than non-severely ill patients (67.4% vs. 23.5%, *p* < 0.01). Most patients received oral antiviral therapy [97.2%, 141/145, (lopinavir/ritonavir, 96.2%, 138/145, or arbidol, 43.4%, 63/145)], atomized inhalation of interferon therapy (96.6%, 140/145), and traditional Chinese medicine treatment (90.3%, 131/145). Many received glucocorticoid therapy (32.4%, 47/145), intravenous immunoglobin therapy (28.3%, 41/145), and antibacterial therapy (19.3%, 28/145). Compared with non-severely ill patients, the proportion of severely ill patients receiving intravenous immunoglobulin therapy and glucocorticoid therapy were higher (83.7% vs. 4.9% and 88.4% vs. 8.8%, both *p* < 0.01), and the course of antiviral treatment was longer (mean, days, 20.29 vs. 15.69, *p* < 0.01). A total of 67.6% (98/145) of the patients received oxygen therapy, and the rate of this in severely ill patients was as high as 100% (*p* < 0.01). Of the 43 severely ill patients, 14% (6/43) received high-flow nasal cannula oxygen therapy, and 2.3% (1/43) received invasive mechanical ventilation. In addition, severely ill patients had prolonged length of hospital stay compared with non-severely ill patients, [median, days, 22(15.5–25.5) vs. 13(9–18), *p* < 0.01].

## Discussion

This report, to our knowledge, is one of the largest case series of patients with COVID-19 in Taizhou, Zhejiang Province, mostly imported from the city of Wuhan.

The severely ill patients were older and had comorbidities such as obesity and diabetes mellitus more often than non-severely ill patients. As with previous studies [21], there were no gender difference between severely and non-severely ill patients in our study. Our study has shown that severely ill patients had a higher APACHE II score and a higher SOFA score. In addition, we also found that severely ill patients had a higher MuLBSTA score than non-severely ill patients. The MuLBSTA score is an early warning model for predicting mortality in viral pneumonia [22]. However, since there are no deaths in our study, further investigation is needed to explore the applicability of the MuLBSTA score in predicting the risk of mortality in COVID-19.

In our study, dry cough and fever were dominant symptoms. Notably, fever occurred in 68.6% of non-severely ill patients and 90.7% severely ill patients. Thus, non-severely ill patients with absence of fever may be missed if the surveillance case definition focused heavily on fever detection. Consistent with previous studies [18, 21, 23], our study also found that the absolute value of lymphocytes in most patients decreased, and it was more obvious in severely ill patients. Severely ill patients were more likely to show prolonged activated partial thromboplastin time, a higher level of troponin I, creatine kinase, aspartate aminotransferase, gamma glutamyl transpeptidase, lactate dehydrogenase and procalcitonin than non-severely ill patients. These results further confirm that pathogenicity of SARS-CoV-2 infection may be associated with cellular immune deficiency, coagulation activation, myocard injury, and hepatic injury [21]. Our study found that in the lung imaging CT of severely ill patients were mostly bilateral pulmonary lesions. This suggests that perhaps bilateral pneumonia is one of the risk factors for severely ill patients.

Until now, all treatments possibilities are still mainly due to meticulous supportive care and improve self-immunity. According to the suggestion of Diagnosis and Treatment of Pneumonia Caused by SARS-CoV-2 (version 7) [19], all of the patients in this study received antiviral therapy (lopinavir/ritonavir or arbidol), aerosol inhalation of interferon-alpha and traditional Chinese medicine treatment. 19.3% received antibiotic therapy, and 28.3% received intravenous immuno-globulin (IVIG). Because severely ill patients are more likely to suffer from lymphopenia, intravenous immunoglobulin have been given to enhance the anti-infection defense reaction of severely ill patients [23]. In addition, among our cohort of 145 confirmed patients with COVID-19, glucocorticoid was given to 8.8% of non-severely ill patients and 88% of severely ill patients. However, the use of glucocorticoid is still controversial. According to WHO interim guidance, glucocorticoid should not be routinely given systemically [16]. Another study has shown that clinical evidence does not support glucocorticoid treatment for COVID-19 and that no benefit was observed from glucocorticoid support [24]. However, according to the study of Jinyintan Hospital [23], it is suggested that steroids (methylprednisolone 1–2 mg/kg per day) are recommended for patients with ARDS, for as short a duration of treatment as possible. Further evidence is urgently needed to assess whether systematic glucocorticoid treatment is beneficial or harmful for patients with COVID-19. 90.3% of patients have been treated with traditional Chinese medicine. In another report [25], traditional Chinese medicine (Shufeng Jiedu Capsule) treatment has also shown a certain improvement of the clinical symptoms.

Our study has some notable limitations. First, most cases were diagnosed in out-patient settings of the local hospital where medical information was briefly documented, and then transferred to our institution for centralized treatment. Some cases had incomplete documentation of symptoms and laboratory testing given the variation in the structure of electronic database among different participating sites. Second, because some critical ill patients were transferred to provincial medical institutions for unified treatment, only one critical ill patient was monitored in our study. Thus, our research may not be applicable to critically ill patients and differences in prevalence of comorbidities might go undetected. Last, we took reference on Diagnosis and Treatment of Pneumonia Caused by SARS-CoV-2 (version 7) [19] issued by of National Health Commission of the People's Republic of China, to define the severity of COVID-19, so its applicability may be limited.

## Conclusions

Older patients or patients with comorbidities such as obesity or diabetes mellitus were more likely to have severe condition. Treatments of COVID-19 is still experimental and more clinical trials are needed.

## Abbreviations

COVID-19: Corona Virus Disease 2019
SARS-CoV-2: Severe acute respiratory syndrome coronavirus 2
SARS: Severe acute respiratory syndrome
CT: Computed tomographic
APACHE: Acute physiology and chronic health evaluation
SOFA: Sequential organ failure assessment
ARDS: Acute respiratory distress syndrome
ICU: Intensive care unit
IQR: Interquartile range
WHO: World Health Organization
RT-PCR: Reverse-transcriptase polymerase-chain-reaction
IVIG: Intravenous immuno-globulin
BMI: Body mass index

## References

[1] Lu H, Stratton CW, Tang YW (2020). Outbreak of pneumonia of unknown etiology in Wuhan, China: The mystery and the miracle. J Med Virol.

[2] Hui DS, Ia E, Madani TA, Ntoumi F, Kock R, Dar O (2020). The continuing 2019-nCoV epidemic threat of novel coronaviruses to global health-The latest novel coronavirus outbreak in Wuhan China. Int J Infect Dis.

[3] Report of novel coronavirus-infected pneumonia in China. Wuhan Municipal Health Commission. 2020. https://wjw.wuhan.gov.cn/front/web/showDetail/2020012009077. Accessed March 28th 2020

[4] Paules CI, Marston HD, Fauci AS (2020). Coronavirus infections-more than just the common cold. JAMA.

[5] Report of clustering pneumonia of unknown etiology in Wuhan City. Wuhan Municipal Health Commission. 2019. https://wjw.wuhan.gov.cn/front/web/showDetail/2019123108989. Accessed March 28th 2020.

[6] Zhu N, Zhang D, Wang W, Li X, Yang B, Song J (2020). A novel coronavirus from patients with pneumonia in China, 2019. N Engl J Med.

[7] Gorbalenya AE, Baker SC, Baric RS, de Groot RJ, Drosten C, Gulyaeva AA (2020). The species Severe acute respiratory syndrome-related coronavirus: classifying 2019-nCoV and naming it SARS-CoV-2. Nature Microbiol.

[8] Chan JF, Yuan S, Kok KH, To KK, Chu H, Yang J (2020). A familial cluster of pneumonia associated with the 2019 novel coronavirus indicating person-to-person transmission: a study of a family cluster. Lancet.

[9] Phan LT, Nguyen TV, Luong QC, Nguyen TV, Nguyen HT, Le HQ (2020). Importation and human-to-human transmission of a novel coronavirus in Vietnam. N Engl J Med.

[10] Rothe C, Schunk M, Sothmann P, Bretzel G, Froeschl G, Wallrauch C (2020). Transmission of 2019-nCoV infection from an asymptomatic contact in Germany. N Engl J Med.

[11] Wu JT, Leung K, Leung GM (2020). Nowcasting and forecasting the potential domestic and international spread of the 2019-nCoV outbreak originating in Wuhan, China: a modelling study. Lancet.

[12] Li Q, Guan X, Wu P, Wang X, Zhou L, Tong Y (2020). Early transmission dynamics in Wuhan, China, of novel coronavirus-infected pneumonia. N Engl J Med.

[13] Guan WJ, Ni ZY, Hu Y, Liang WH, Ou CQ, He JX (2020). Clinical characteristics of coronavirus disease 2019 in China. N Engl J Med.

[14] National Health Commission of the People’s Republic of China. https://www.nhc.gov.cn/xcs/yqtb/202004/82ca3a872c864abc80a538c0ec948f10.shtml. Accessed April 14th 2020. 10.46234/ccdcw2020.082 PMC8392946 34594648

[15] Coronavirus disease (COVID-2019) situation reports. World Health Organization. https://www.who.int/emergencies/diseases/novel-coronavirus-2019/situation-reports. Accessed April 14th 2020.

[16] Clinical management of severe acute respiratory infection when COVID-19 is suspected. (2020) World Health Organization. https://www.who.int/publications-detail/clinical-management-of-severe-acute-respiratory-infection-when-novel-coronavirus-(ncov)-infection-is-suspected. Accessed April 9th 2020.

[17] Huang C, Wang Y, Li X, Ren L, Zhao J, Hu Y (2020). Clinical features of patients infected with 2019 novel coronavirus in Wuhan China. Lancet.

[18] Force ADT, Ranieri VM, Rubenfeld GD, Thompson BT, Ferguson ND, Caldwell E (2012). Acute respiratory distress syndrome: the Berlin Definition. JAMA.

[19] Diagnosis and Treatment of Pneumonia Caused by SARS-COV-2 (version 7). National Health Commission of the People’s Republic of China. https://www.nhc.gov.cn/xcs/zhengcwj/202003/46c9294a7dfe4cef80dc7f5912eb1989/files/ce3e6945832a438eaae415350a8ce964.pdf. Accessed April 9th 2020.

[20] Singer M, Deutschman CS, Seymour CW, Shankar-Hari M, Annane D, Bauer M (2016). The third international consensus definitions for sepsis and septic shock (Sepsis-3). JAMA.

[21] Wang D, Hu B, Hu C, Zhu F, Liu X, Zhang J (2020). Clinical characteristics of 138 hospitalized patients with, novel coronavirus-infected pneumonia in Wuhan, China. JAMA.

[22] Guo L, Wei D, Zhang X, Wu Y, Li Q, Zhou M (2019). Clinical features predicting mortality risk in patients with viral pneumonia: The MuLBSTA Score. Front Microbiol.

[23] Chen N, Zhou M, Dong X, Qu J, Gong F, Han Y (2020). Epidemiological and clinical characteristics of 99 cases of 2019 novel coronavirus pneumonia in Wuhan, China: a descriptive study. Lancet.

[24] Russell CD, Millar JE, Baillie JK (2020). Clinical evidence does not support corticosteroid treatment for 2019-nCoV lung injury. The Lancet.

[25] Wang Z, Chen X, Lu Y, Chen F, Zhang W (2020). Clinical characteristics and therapeutic procedure for four cases with 2019 novel coronavirus pneumonia receiving combined Chinese and Western medicine treatment. Biosci Trends.

